# Target-controlled infusions of butorphanol worsen hemodynamics in isoflurane-anesthetized cats

**DOI:** 10.3389/fvets.2025.1600753

**Published:** 2025-06-03

**Authors:** Bruno H. Pypendop, Alessia Cenani, Marcela L. Machado, Linda S. Barter

**Affiliations:** Department of Surgical and Radiological Sciences, School of Veterinary Medicine, University of California, Davis, Davis, CA, United States

**Keywords:** cats, anesthesia, butorphanol, isoflurane, hemodynamics

## Abstract

**Introduction:**

Partial intravenous anesthesia can be used to reduce the adverse effects of inhalant anesthetics, and opioids are often used in this context in dogs and cats. The opioid butorphanol was recently shown to dose-dependently reduce the minimum alveolar concentration of isoflurane in cats. The aim of this study was to characterize the hemodynamic effects of butorphanol in isoflurane-anesthetized cats.

**Methods:**

Six cats were anesthetized with isoflurane and administered butorphanol to maintain target plasma concentrations (TPCs) of 0, 1.1, 2.2 and 4.4 μg mL^−1^, with or without concurrent administration of atropine. The isoflurane concentration was adjusted to maintain equipotency based on the results of a previous study. Cardiorespiratory variables were measured at each plasma butorphanol concentration.

**Results:**

Severe bradycardia and hypotension were observed in one cat at the 4.4 μg mL^−1^ TPC and in one cat at the 2.2 μg mL^−1^ TPC without atropine. Treatments without atropine and the 4.4 μg mL^−1^ TPC were not studied in subsequent cats. Butorphanol significantly reduced heart rate, cardiac index and oxygen delivery index, and increased systemic vascular resistance index and oxygen consumption index in a plasma concentration-dependent manner.

**Discussion:**

In isoflurane-anesthetized cats, at the TPCs studied, butorphanol caused more cardiovascular depression than a higher, equipotent concentration of isoflurane alone.

## 1 Introduction

Partial intravenous anesthesia is used to decreased the concentration of inhaled anesthetics required to produce anesthesia through the concurrent use of injectable drugs (1). The primary goals are to reduce the adverse effects induced by inhaled anesthetics, including the cardiovascular depression, provide analgesia and improve anesthetic quality. Opioids are commonly used for partial intravenous anesthesia in dogs and cats (1). However, several studies in cats have shown mu opioid receptor agonists produce moderate to no reduction in inhaled anesthetic requirements, limiting their benefits (2–6). The largest reduction in inhaled anesthetic minimum alveolar concentration (MAC) induced by a mu opioid receptor agonist reported in cats is 35%, less than half the value reported in dogs (4, 7, 8).

Butorphanol is an agonist of kappa opioid receptors and an antagonist of mu opioid receptors (9). A recent study in cats showed it reduced the MAC of isoflurane in a dose-dependent manner by up to 68%, with a linear dose-response relationship within the range of doses studied (10). Only limited cardiorespiratory measurements were obtained in that study.

In the previous study, the higher dose of butorphanol studied caused a significant reduction in pulse rate (10). Opioid-induced bradycardia can be treated with anticholinergics such as atropine (11).

The aim of this study was to characterize the hemodynamic effects of butorphanol in isoflurane-anesthetized cats. It was hypothesized that butorphanol would decrease heart rate in a plasma concentration-dependent manner, that atropine would counteract this effect, and that cardiac index would be higher during butorphanol administration than with an equipotent concentration of isoflurane alone when atropine was given.

## 2 Materials and methods

### 2.1 Animals

A group of six 4 to 9 years old male neutered domestic shorthair purpose-bred research cats of 4.7 ± 0.3 kg (mean ± standard deviation) were used. The study was approved by the Institutional Animal Care and Use Committee of UC Davis (protocol 22426). Inclusion of cats required normal health based on lack of history of disease and abnormal findings on physical examination before the study. No additional tests were completed. There were no exclusion criteria. Two cats were housed together in a room. The remaining four cats were housed individually in cages due to aggressive behavior towards other cats. The room and cages were equipped with shelves for climbing, toys and cardboard boxes for hiding. The rooms in which cats were kept were on a 12:12 h light:dark cycle. Housing conditions were within the range required by the Animal Welfare Act and Animal Welfare Regulations of the United States Department of Agriculture (https://www.aphis.usda.gov/sites/default/files/AC_BlueBook_AWA_508_comp_version.pdf; accessed on November 13, 2024). Cats were fed commercial dry food (Laboratory Feline Diet 5003, LabDiet, MO, United States) once a day and had access to water *ad libitum*. Cats were fasted for a minimum of 12 h prior to each experiment but continued to have access to water until they were moved to a laboratory for the experiment approximately 30 min before anesthesia commenced.

### 2.2 Instrumentation

Cats were placed in an acrylic chamber into which isoflurane was delivered in oxygen via a Bain breathing system. The isoflurane vaporizer was set at 5% and the oxygen flow at 5 L minute^−1^. After the righting reflex was lost, cats were removed from the chamber. Anesthesia was deepened as necessary to allow intubation of the trachea by applying a tight-fitting facemask connected to the Bain breathing system to deliver 5% isoflurane in 2 L minute^−1^ oxygen. The trachea was intubated with a cuffed 4.5 mm internal diameter tube and the cuff was inflated as to prevent an audible gas leak around the tube during positive pressure ventilation at a pressure of 15 cm H_2_O. A surgical depth of anesthesia, characterized by mild tone of the jaw muscles, eccentric eye position and absence of the palpebral reflex, was maintained with isoflurane delivered in a minimum of 200 mL kg^−1^ min^−1^ oxygen, using the Bain breathing system. A 22-gauge, 4.4 cm catheter (Introcan Safety IV Catheter, B Braun, PA, United States) was placed in a cephalic vein for administration of the study drugs and lactated Ringer’s solution (LRS; Baxter Healthcare Corp., IL, United States) at 3 mL kg^−1^ h^−1^. Cefazolin (25 mg kg^−1^, Cefazolin Sodium, Sagent Pharmaceuticals Inc., IL, United States) was given intravenously as prophylaxis for carotid artery cutdown and/or cardiac catheterization. A 22-gauge, 4.4 cm catheter was placed in the carotid artery via cutdown (first experiment only) or in the ventral coccygeal artery. If placement in the coccygeal artery was not successful, a 22-gauge, 12 cm arterial catheter (Teleflex, NC, United States) was inserted in the femoral artery under ultrasound guidance. The arterial catheter was used for arterial pressure measurement and blood sampling for blood gas and drug concentration analysis. A 5-Fr, 7.5 cm introducer (Arrow Percutaneous Sheath Introducer Set with Integral Hemostasis Valve/Side Port, Teleflex, NC, United States) was inserted in an external jugular vein. A 5-Fr, 78 cm thermodilution catheter (Swan-Ganz Pulmonary Thermodilution Catheter, Edwards Lifesciences, CA, United States) was inserted through the introducer and the distal port and thermistor were positioned in the pulmonary artery under fluoroscopic guidance.

### 2.3 Measurements

Cats were placed in lateral recumbency. Anesthesia was maintained with isoflurane in oxygen using a circle breathing system and mechanical ventilation (Carestation 620, GE Healthcare, WI, United States). The mechanical ventilator was set in the pressure control/volume guaranteed mode with a tidal volume of 10–12 mL kg^−1^ and adjusting the respiratory rate to maintain the end-tidal partial pressure of carbon dioxide (PCO_2_) within 35–45 mm Hg range. End-tidal PCO_2_ was measured using an infrared spectrophotometer (Carescape B650, GE Healthcare, WI, United States) that was sampling gas from a catheter inserted in the endotracheal tube so that the catheter tip was near the tracheal end of the tube. End-tidal isoflurane concentration (ETiso) was measured in samples hand-collected from the same catheter in glass syringes, using the same infrared spectrophotometer. Three samples were collected and the mean ETiso was calculated and considered the ETiso for that measurement. The accuracy of the spectrophotometer was verified by measurement of isoflurane concentration in room air and in a secondary standard with a known isoflurane concentration of 3.33%. A lead II electrocardiogram was continuously recorded using a data acquisition system (PowerLab 4 and LabChart, ADInstruments, CO, United States). Heart rate (HR) was obtained from the electrocardiogram. The arterial catheter and the proximal and distal ports of the thermodilution catheter were connected to transducers (Meritrans DTXPlus, Merit Medical, UT, United States) using saline-filled noncompliant tubing for continuous recording of mean arterial pressure (MAP; arterial catheter), central venous pressure (proximal thermodilution catheter port) and mean pulmonary artery pressure (distal port of the thermodilution catheter) using the same data acquisition system. Transducers were calibrated daily across a range of pressures encompassing the measurements (0 and 250 mm Hg for arterial pressure, 0 and 40 mm Hg for pulmonary artery pressure and 0 and 25 mm Hg for central venous pressure) against a mercury column, were zeroed to atmospheric pressure and leveled with the sternum (with the cats in lateral recumbency) for the measurements. The position of the thermodilution catheter was adjusted as needed until a typical pulmonary artery waveform was observed in the recording from the distal port of the catheter. Pulmonary artery occlusion pressure was measured at selected time points from the distal port of the thermodilution catheter by inflating the balloon of the catheter. Body temperature was measured using the thermistor of the thermodilution catheter and maintained between 37.5 and 38.5°C by providing external heat (Hotdog and/or Bair Hugger, Augustine Medical Inc., MN, United States) as needed. Arterial and mixed-venous (i.e., pulmonary artery) blood samples (0.5 mL) were simultaneously collected in syringes containing dry heparin (safePICO Aspirator, Radiometer America, CA, United States) over approximately 5 s to measure arterial and mixed-venous hemoglobin concentration, hemoglobin oxygen saturation (SaO_2_ and SvO_2_), pH, and partial pressure of oxygen (PaO_2_ and PvO_2_) and carbon dioxide (PaCO_2_ and PvCO_2_) using a blood gas machine (ABL 820*flex*, Radiometer America). Cardiac output was measured by injection of 3 mL of cold (0–5°C) dextrose 5% in water (Baxter Healthcare Corp.) through the proximal port of the thermodilution catheter and use of a cardiac output computer (Carescape B650). Measurements were repeated until 3 measurements within 10% of each other were obtained. Additional cardiorespiratory variables were calculated from the measurements using previously published equations (12). Arterial blood samples (2 mL) were collected prior to treatment administration and 45 min after changing the target plasma butorphanol concentration. Samples were transferred to tubes containing ethylenediaminetetraacetic acid and, within 10 min of collection, centrifuged at 3,901 g and 4°C for 10 min. The plasma was separated, transferred in cryotubes and frozen at −80°C until analysis for butorphanol concentration using liquid chromatography/tandem mass spectrometry as previously described (13, 14). The limit of quantitation of the assay was 1 ng mL^−1^ and the accuracy and imprecision were 94–105% and 2–4%, respectively.

### 2.4 Treatments

Following instrumentation, ETiso was set at 1.25 times the MAC reported in a previous study on butorphanol in cats (2.48%) (10). After 20 min at that ETiso, HR, MAP, mean pulmonary artery pressure, pulmonary artery occlusion pressure and body temperature were recorded. Arterial and mixed venous blood samples were collected, and cardiac output was measured. The measurements were repeated at 40 min. Following completion of the measurements, intravenous administration of butorphanol (Torbugesic, Zoetis, MI, United States) was started using a target-controlled infusion, using published pharmacokinetic parameters for butorphanol in isoflurane-anesthetized cats (14). The target-controlled infusion system comprised a syringe pump (PHD 2000, Harvard Apparatus, MA, United States) and computer software (Rugloop I, Demed, Belgium). The target plasma butorphanol concentration (TPC) was set at 1.1 μg mL^−1^. ETiso was reduced to 1.91%, corresponding to 1.25 times the MAC reported at that plasma butorphanol concentration in a previous study (10). Measurements were obtained as described above 20 and 45 min after starting butorphanol administration. Following completion of the measurements, the TPC was increased to 2.2 μg mL^−1^, ETiso was reduced to 1.58%, corresponding to 1.25 times the MAC reported at that plasma butorphanol concentration in the previous study and measurements were repeated as described above. Finally, following completion of the measurements, the TPC was increased to 4.4 μg mL^−1^, ETiso was reduced to 0.8%, corresponding to 1.25 times the MAC reported at that plasma butorphanol concentration in the previous study and the measurements repeated. After the last measurement, instruments were removed. Pressure was applied for 10 min over the jugular introducer and arterial catheter sites. Meloxicam (0.1 mg kg^−1^ subcutaneous; Ostilox Injection, Vet One MWI, ID, United States) was given. In addition, if a carotid catheter had been placed via cutdown, the site was closed in two layers (subcutaneous and subcuticular) and buprenorphine (20 μg kg^−1^ intravenously, Buprenorphine HCl, Par Pharmaceuticals, NY, United States) was also given. Cats were observed until recovered from anesthesia and able to walk normally. They were then returned to their normal housing and assessed at least once a day for 10 days for signs of pain or any other abnormality observed on physical examination.

Cats were studied twice, once without, and once with atropine (Atropine Sulfate Injection, USP, Hikma, NJ, United States) titrated intravenously in 0.01 mg kg^−1^ increments to maintain heart rate at 150 beats minute^−1^ or above or until no effect on heart rate was detected, whichever occurred first. The order of treatments (atropine or no atropine) was randomized using an online randomizer.^1^

### 2.5 Statistical analysis

Power analysis based on a previous study (15) suggests that six cats would allow to detect a 20% treatment effect on cardiac output and mean arterial pressure with a power of 0.8 and the alpha level set at 0.05.

Normality of the data was verified by the Shapiro–Wilk test. Data that were not normally distributed were log-transformed for analysis. A two-way repeated measures analysis of variance was used to analyze the effects of treatment, time and their interaction, with the cat set as a random factor. Where appropriate, pairwise treatment comparison were conducted using the Tukey test, and pairwise time comparison within treatment were conducted using Student’s *t*-test. The alpha level was set at 0.05. All analyses were conducted using commercial software (JMP Pro 17, SAS Institute, NC, United States and Prism 10, GraphPad, CA, United States). Normally distributed data are presented as mean ± standard deviation and non-normally distributed data are presented as median (interquartile range).

## 3 Results

After 14 min of infusion at the 4.4 μg mL^−1^ TPC (no atropine), the first cat’s HR was 67 beats minute^−1^, and its MAP was 35 mm Hg. The cat was removed from the study protocol, the infusion of butorphanol and isoflurane administration were discontinued, and atropine (0.02 mg kg^−1^) was given intravenously. The cat remained comatose and was transferred to the intensive care unit. Twenty-four hours later, minimal improvement in mentation was observed and the cat was euthanized by intravenous injection of 100 mg kg^−1^ of pentobarbital (Euthasol, Virbac, TX, United States). A necropsy was performed and revealed severe diffuse brain edema and encephalitis, affecting the cerebrum more than the brainstem. No significant other abnormalities were found. Given the severity of the bradycardia and hypotension at the 4.4 μg mL^−1^ TPC, it was decided to not include this treatment in the subsequent cats. Two other cats were randomized to not receive atropine in the first arm of the study. Both developed bradycardia (HR <120 beats minute^−1^) and hypotension (MAP <60 mm Hg) at both TPCs of 1.1 and 2.2 μg mL^−1^. In the second of these cats, after 10 min of infusion at the 2.2 μg mL^−1^ TPC, HR was 56 beats minute^−1^ and MAP was 32 mm Hg. The cat was removed from the study protocol and the isoflurane concentration was reduced with no improvement. Isoflurane administration and butorphanol infusion were then ceased, and atropine (0.04 mg kg^−1^), naloxone (0.04 mg kg^−1^) and LRS (50 mL) were given intravenously. Heart rate and MAP normalized within 3 min and the cat recovered uneventfully. Because of the severity of the bradycardia and hypotension and the low likelihood that butorphanol infusions at the doses studied without concurrent administration of atropine would be clinically useful, the remaining studies without atropine were cancelled. Therefore, the results presented include five cats for the 0, 1.1 and 2.2 μg mL^−1^ TPCs with concurrent administration of atropine only.

Isoflurane concentration was 101 ± 2% of the desired concentration (all measurements pooled). Plasma butorphanol concentration was 0 ± 0, 1.02 ± 0.08 and 2.50 ± 0.22 μg mL^−1^ for the 0, 1.1 and 2.2 μg mL^−1^ TPCs, respectively. The total dose of atropine was 0 (0), 0.04 (0.03) and 0.02 (0.02) mg kg^−1^ for the 0, 1.1 and 2.2 μg mL^−1^ TPCs, respectively. The target heart rate (150 beats minute^−1^) was only achieved in one cat at 20 min in the 1.1 μg mL^−1^ TPC treatment.

Measured physiological data, results of blood gas analysis and calculated physiologic data are presented in Tables 1–3, respectively. Heart rate, arterial and mixed-venous pH, arterial bicarbonate concentration, arterial standard base excess, PvO_2_, SvO_2_, cardiac index (CI), stroke index, left ventricular stroke work index, right ventricular stroke work index, rate-pressure product, mixed-venous oxygen concentration, oxygen delivery index and shunt fraction significantly decreased with increasing TPCs. Central venous pressure, mean pulmonary artery pressure, systemic vascular resistance index (SVRI), arterial oxygen concentration, oxygen consumption index and oxygen utilization ratio significantly increased with increasing TPCs. The target plasma butorphanol concentration did not significantly affect MAP, pulmonary artery occlusion pressure, body temperature, PaO_2_, PaCO_2_, PvCO_2_, SaO_2_, mixed-venous bicarbonate concentration, mixed-venous standard base excess, pulmonary vascular resistance index or the alveolar-to-arterial PO_2_ difference.

**Table 1:** Mean ± standard deviation or median (interquartile range) of the measured physiological variables in 5 isoflurane-anesthetized cats during mechanical ventilation and administration of butorphanol during a target-controlled infusion to achieve and maintain plasma butorphanol concentrations of 0, 1.1 and 2.2 µg mL^−1^.

Butorphanol TPC	0 μg mL^−1^		1.1 μg mL^−1^		2.2 μg mL^−1^	
Time (minutes)	20	45	20	45	20	45
Atropine (mg kg^−1^)	0 (0)		0.04 (0.03)		0.02 (0.02)	
HR (beats minute^−1^)^a^	158 (7)	156 (10)	140 (22)^b^	137 (17)^b^	122 (16)^b^^,^^c^	119 (13)^b^^,^^c^
MAP (mm Hg)	70 ± 14	64 ± 11	73 ± 13	66 ± 9	68 ± 8	61 ± 10
CVP (mm Hg)^a^	5 ± 1	5 ± 1	6 ± 1	6 ± 1	9 ± 1^b^^,^^c^	9 ± 1^b^^,^^c^
MPAP (mm Hg)^a^	13 ± 1	13 ± 1	14 ± 2	14 ± 2	15 ± 1^b^	15 ± 1^b^
PAOP (mm Hg)	5 (5)	5 (6)	5 (6)	6 (7)	7 (6)	7 (3)
Body temperature (°C)	38.2 ± 0.2	38.3 ± 0.1	38.2 ± 0.2	38.2 ± 0.2	38.2 ± 0.1	38.3 ± 0.1

TPC, target plasma concentration; HR, heart rate; MAP, mean arterial pressure; CVP, central venous pressure; MPAP, mean pulmonary artery pressure; PAOP, pulmonary artery occlusion pressure. Significance was set at *p* < 0.05. *p*-values are reported in the Supplementary material. Note that atropine was administered prior to the 20 min measurement.

a Significant effect of the TPC.

b Significantly different from TPC of 0 μg mL^−1^.

c Significantly different from TPC of 1.1 μg mL^−1^.

**Table 2:** Mean ± standard deviation or median (interquartile range) blood gas analysis data in 5 isoflurane-anesthetized cats during mechanical ventilation and administration of butorphanol using a target-controlled infusion to achieve and maintain plasma butorphanol concentrations of 0, 1.1 and 2.2 μg mL^−1^.

Butorphanol TPC	0 μg mL^−1^		1.1 μg mL^−1^		2.2 μg mL^−1^	
Time (minutes)	20	45	20	45	20	45
Arterial pH^a^^,^^b^^,^^c^	7.38 (0.04)	7.35 (0.05)^f^	7.33 (0.02)	7.34 (0.01)	7.33 (0.02)^d^	7.33 (0.03)^d^
PaO_2_ (mm Hg)	430 ± 71	459 ± 38	471 ± 45	449 ± 38	474 ± 38	449 ± 19
PaCO_2_ (mm Hg)^b^^,^^c^	34.3 ± 3.5	36.4 ± 4	37.3 ± 2.8	35.7 ± 3.5	36.5 ± 3.3	36.4 ± 4.1
Arterial HCO_3_^−^ (mEq L^−1^)^a^	19.5 ± 0.9	19.4 ± 1.9	19.4 ± 1.3	18.9 ± 1.7	18.8 ± 1.2^d^^,^^e^	18.4 ± 1.4^d^^,^^e^
Arterial SBE (mEq L^−1^)^a^	−4.4 ± 0.8	−4.7 ± 2	−4.9 ± 1.3	−5.3 ± 1.7	−5.5 ± 1.1^d^	−6 ± 1.3^d^
SaO_2_ (%)	100 (0)	100 (0)	100 (0)	100 (0)	100 (0)	100 (0)
MV pH^a^^,^^b^	7.32 ± 0.02	7.3 ± 0.02	7.28 ± 0^d^	7.28 ± 0.01^d^	7.26 ± 0.01^d^	7.25 ± 0.01^d^
PvO_2_ temp (mm Hg)^a^	70 ± 6	70 ± 7	61 ± 8^d^	60 ± 6^d^	46 ± 5^d^^,^^e^	46 ± 4^d^^,^^e^
PvCO_2_ (mm Hg)	42.6 ± 2.7	43.3 ± 3.3	44.6 ± 3	44.6 ± 2.8	46.2 ± 2.1	48.5 ± 2.4
MV HCO_3_^−^ (mEq L^−1^)	21 ± 0.8	20.6 ± 1.3	20.3 ± 1.5	20.4 ± 1.4	20.3 ± 1	20.7 ± 0.5
MV SBE (mEq L^−1^)	−3.5 ± 0.7	−4.1 ± 1.4	−4.6 ± 1.6	−4.4 ± 1.4	−4.8 ± 1	−4.5 ± 0.4
SvO_2_ (%)^a^	92 (3)	91 (4)	83 (9)	83 (8)	69 (10)^d^^,^^e^	66 (10)^d^^,^^e^

TPC, target plasma concentration; PaO_2_, arterial partial pressure of oxygen; PaCO_2_, arterial partial pressure of carbon dioxide; HCO_3_^−^, plasma bicarbonate concentration; SBE, standard base excess; SaO_2_, arterial hemoglobin oxygen saturation; MV, mixed venous; PvO_2_, mixed venous partial pressure of oxygen; PvCO_2_, mixed venous partial pressure of carbon dioxide; SvO_2_, mixed venous hemoglobin oxygen saturation. Significance was set at *p* < 0.05. *p*-values are reported in the Supplementary material.

a Significant effect of the TPC.

b Significant effect of time.

c Significant TPC × time interaction.

d Significantly different from TPC of 0 μg mL^−1^.

e Significantly different from TPC of 1.1 μg mL^−1^.

f Significantly different from 20 min.

**Table 3:** Mean ± standard deviation or median (interquartile range) of the calculated physiologic data in 5 isoflurane-anesthetized cats during mechanical ventilation and administration of butorphanol using a target-controlled infusion to achieve and maintain plasma butorphanol concentrations of 0, 1.1 and 2.2 μg mL^−1^.

Butorphanol TPC	0 μg mL^−1^		1.1 μg mL^−1^		2.2 μg mL^−1^	
Time (minutes)	20	45	20	45	20	45
CI (L minute^−1^ BW^−0.67^)^a^	0.22 ± 0.03	0.22 ± 0.02	0.18 ± 0.01^d^	0.17 ± 0.01^d^	0.12 ± 0.01^d^^,^^e^	0.12 ± 0.01^d^^,^^e^
SI (mL beat^−1^ kg^−1^)^a^^,^^c^	0.83 ± 0.11	0.83 ± 0.09	0.79 ± 0.04	0.74 ± 0.04	0.62 ± 0.06^d^^,^^e^	0.63 ± 0.06^d^^,^^e^
SVRI (dynes seconds cm^−5^ BW^−0.67^)^a^	24,233 ± 7,336	21,894 ± 5,430	29,287 ± 4,348	28,500 ± 3,167	38,634 ± 4,067^d^	33,992 ± 3,532^d^
PVRI (dynes seconds cm^−5^ BW^−0.67^)	2,559 ± 914	2,526 ± 1,025	3,276 ± 1,298	3,375 ± 1,373	4,334 ± 2,229	5,345 ± 1,411
LVSWI (cJ kg^−1^)^a^	0.65 (0.25)	0.57 (0.17)	0.67 (0.3)	0.56 (0.2)	0.56 (0.2)^d^	0.44 (0.19)^d^
RVSWI (cJ kg^−1^)^a^	0.08 ± 0.01	0.09 ± 0.01	0.08 ± 0.01	0.08 ± 0.01	0.05 ± 0^d^^,^^e^	0.05 ± 0.01^d^^,^^e^
RPP (beats minute^−1^ mm Hg)^a^^,^^b^	15,832 ± 2,144	14,032 ± 1,770^f^	13,714 ± 1,688	12,165 ± 1,568	11,396 ± 1,713^d^	10,286 ± 1,922^d^
CaO_2_ (mL dL^−1^)^a^	11.3 ± 1.3	11 ± 1.4	11.1 ± 1.4	10.9 ± 1.6	12.2 ± 1.9^d^^,^^e^	12 ± 1.8^d^^,^^e^
CvO_2_ (mL dL^−1^)^a^	9.2 ± 1.4	8.8 ± 1.3	7.5 ± 1.5^d^	7.3 ± 1.6^d^	6.1 ± 1.4^d^^,^^e^	6.1 ± 1.1^d^^,^^e^
DO_2_I (mL minute^−1^ BW^−0.67^)^a^	24.5 ± 2.4	24 ± 2	20 ± 3.3^d^	18.2 ± 3.6^d^	15 ± 2.2^d^^,^^e^	14.5 ± 1.6^d^^,^^e^
VO_2_I (mL minute^−1^ BW^−0.67^)^a^	4.6 ± 1.9	4.9 ± 1.2	6.5 ± 1	6 ± 0.3	7.6 ± 0.8^d^	7.1 ± 1.1^d^
Oxygen utilization ratio^a^	0.18 ± 0.07	0.2 ± 0.05	0.33 ± 0.07^d^	0.34 ± 0.06^d^	0.51 ± 0.07^d^^,^^e^	0.49 ± 0.08^d^^,^^e^
Qs/Qt^a^	0.22 (0.26)	0.22 (0.12)	0.15 (0.08)^d^	1.16 (0.04)^d^	0.09 (0.03)^d^^,^^e^	0.11 (0.04)^d^^,^^e^
P(A-a)O_2_ (mm Hg)	251 ± 72	221 ± 38	208 ± 46	231 ± 41	205 ± 42	231 ± 23

TPC, target plasma concentration; CI, cardiac index; BW, body weight; SI, stroke index; SVRI, systemic vascular resistance index; PVRI, pulmonary vascular resistance index; LVSWI, left ventricular stroke work index; RVSWI, right ventricular stroke work index; RPP, rate-pressure product; CaO_2_, arterial oxygen concentration; CvO_2_, mixed venous oxygen concentration; DO_2_I, oxygen delivery index; VO_2_I, oxygen consumption index; Qs/Qt, shunt fraction; P(A-a)O_2_, alveolar-to-arterial difference in partial pressure of oxygen. Significance was set at *p* < 0.05. *p*-values are reported in the Supplementary material.

a Significant effect of the TPC.

b Significant effect of time.

c Significant TPC × time interaction.

d Significantly different from TPC of 0 μg mL^−1^.

e Significantly different from TPC of 1.1 μg mL^−1^.

f Significantly different from 20 min.

A significant effect of time was detected for mixed-venous pH and for the rate-pressure product. A significant effect of time and TPC × time interaction was detected for arterial pH and PaCO_2_. A significant TPC × time interaction was detected for stroke index.

Cats salivated excessively and were not interested in food for a minimum of 24 h and up to 48 h following recovery from anesthesia.

## 4 Discussion

In this study in isoflurane-anesthetized cats, increasing plasma butorphanol concentrations produced cardiovascular depression despite the decrease in MAC produced, characterized by a decrease in HR and CI, an increase SVRI and no significant change in MAP. Atropine was ineffective at producing the target heart rate of 150 beats minute^−1^ during butorphanol infusion and did not prevent a concentration-dependent reduction in HR.

Isoflurane was set at 1.25 times its MAC at the corresponding plasma butorphanol concentrations in a previous study (10). This multiple was selected to represent concentrations resulting in anesthesia in most subjects (16). The plasma butorphanol concentrations used were based on a previous study showing a concentration-dependent decrease in isoflurane MAC (10). Mean plasma butorphanol concentrations in this study were 1.02 and 2.50 μg mL^−1^ for the 1.1 and 2.2 μg mL^−1^ concentrations, compared to 1.17 and 2.20 μg mL^−1^ in the previous study. This indicates that the pharmacokinetic model used for target-controlled infusion was not completely predictive of the actual drug disposition in these cats. More importantly, based on the previous study, the isoflurane concentrations used corresponded to 1.24 and 1.32 MAC in the 1.1 and 2.2 μg mL^−1^ TPC, respectively. Therefore, the results should be interpreted in the context of a moderately higher than desired MAC multiple in the 2.2 μg mL^−1^ TPC.

Butorphanol caused a plasma concentration-dependent decrease in HR in agreement with previous studies in dogs and cats (10, 17–19). In dogs, opioids increase parasympathetic tone and inhibit sympathetic tone, resulting in decreases in HR (20). Because of this mechanism, opioid-induced bradycardia is responsive to anticholinergics such as atropine or glycopyrrolate (20). This was demonstrated in enflurane- and isoflurane-anesthetized dogs receiving high doses of fentanyl (21, 22). However, in isoflurane-anesthetized cats, a high dose of the opioid alfentanil was reported to increase HR via sympathetic stimulation (23). The difference in effects on HR between butorphanol and alfentanil may be related to their receptor effects, with alfentanil being a full *μ*-opioid receptor agonist and butorphanol a *κ*-opioid receptor agonist and μ-opioid receptor antagonist (24, 25). The ineffectiveness of atropine to increase HR to 150 beats minute^−1^ and to prevent butorphanol-induced decrease in HR was not anticipated. A similar observation was made in dogs, although during infusions of very high doses of butorphanol (18). Nevertheless, atropine may have contributed to the prevention of severe bradycardia as the lowest HR observed with atropine treatment was 99 beats minute^−1^ whereas it was 56 at the time of discontinuation of the study in one cat without atropine treatment. Moreover, in that cat, atropine appeared to help normalizing HR after discontinuation of the butorphanol infusion (but at a time at which plasma butorphanol concentration was still expected to be high). Mean HR was lower than 140 beats minute^−1^ at both butorphanol TPCs, a value commonly considered to represent bradycardia in cats (26).

The decrease in HR was partly responsible for the decrease in CI; however, stroke index also decreased. The mechanism for the decrease in stroke index is unclear; it is possible that it is related to an increase in afterload since SVRI increased. However, changes in preload and/or myocardial contractility cannot be ruled out. The effects of opioids on SVRI vary among drugs, with methadone, an agonist of mu opioid receptors with a low affinity for kappa opioid receptors reported to increase SVRI due to release of vasopressin (27–29). A previous study on butorphanol in anesthetized dogs did not find significant changes in SVRI (30). However, a single, moderate bolus dose was used in that study, compared with infusions targeting higher plasma concentrations in the present study.

Central venous pressure was significantly higher at the 2.2 μg mL^−1^ TPC compared to 0 and 1.1 μg mL^−1^. This may be related to the decrease in cardiac index (31). Central venous pressure increases when cardiac index decreases due to a decrease in the volume of blood transferred from the venous to the arterial circulation.

Left and right ventricular stroke work indices decreased with increasing plasma butorphanol concentration, suggesting cardiac work decreased. Similarly, rate-pressure product, an estimate of myocardial oxygen consumption, decreased with increasing plasma butorphanol concentrations. This decrease was primarily due to the decrease in heart rate.

Oxygen delivery index decreased with increasing plasma butorphanol concentrations related to the decrease in CI. Oxygen consumption index increased with increasing plasma butorphanol concentrations. The reasons for this increase are unclear. Isoflurane has been shown to reduce oxygen consumption in a concentration-dependent manner (32). It is therefore possible that the butorphanol-induced reduction in isoflurane concentration resulted in an increase in oxygen consumption index. The combined effects on oxygen delivery index and oxygen consumption index resulted in decreases in PvO_2_, SvO_2_ and mixed venous oxygen concentration and in an increase in oxygen utilization ratio.

Shunt fraction significantly decreased with increasing plasma butorphanol concentrations. This is likely related to the decrease in CI causing a reduction in blood flow through unventilated areas of the lung (33).

Additional variables including mean pulmonary artery pressure, arterial and mixed venous pH, PaCO_2_, arterial bicarbonate concentration and standard base excess and arterial oxygen concentration were significantly affected by butorphanol infusions; however, the changes are of small magnitude and considered clinically insignificant.

The first cat remained comatose at the end of the first experiment (no atropine). This cat experienced severe bradycardia and hypotension but also had an arterial catheter placed in the carotid artery. It is unclear if the neurologic impairment was related to cerebral ischemia secondary to poor perfusion or to arterial embolization. Extreme care was taken to avoid injection of air bubbles through the carotid catheter, and injection of air or other materials was not observed but cannot be completely ruled out. Due to the possibility of arterial embolization, arterial catheters were not placed in the carotid artery in subsequent experiments.

The inappetence and excessive salivation observed for 24–48 h after the study are likely due to the combined effects of butorphanol and atropine on the gastrointestinal system. Both butorphanol and atropine have been reported to decrease gastrointestinal motility (34, 35).

The results of this study should be interpreted in view of several limitations. The sample size was small, and power was calculated only for changes of clinical significance in CI and MAP. It is therefore possible that the study was underpowered for other variables. Moreover, the number of cats used was one less than the sample size calculated by power analysis due to euthanasia of the first cat. Only male neutered cats were used. However, given the small sample studied, it is unlikely that sex differences could have been detected. In addition, sex differences in the effects of opioids have not been reported in cats. Health status determination relied on physical examination and lack of historical evidence of disease. No additional screening test was conducted. Subclinical health abnormalities can therefore not be excluded. Butorphanol was administered using a target-controlled infusion, which is not representative of current clinical practice. However, target-controlled infusions can rapidly achieve and maintain stable plasma drug concentrations. The concentrations achieved were similar but not identical to those in a previous study characterizing the effects of butorphanol on isoflurane MAC (10). This resulted in the isoflurane concentration at the 2.2 μg mL^−1^ TPC to be higher than the 1.25 MAC target. Moreover, individual MAC values were not measured for the cats in this study; rather, values from a previous study were used. The study design included treatments with and without concurrent administration of atropine; however, the treatments without atropine were abandoned due to severe hypotension and bradycardia in two cats. Moreover, a 4.4 μg mL^−1^ was included in the study plan but abandoned after the first cat developed severe hypotension and bradycardia at that concentration. Although it is possible that a majority of cats would have tolerated these treatments, we deemed their likely clinical usefulness to be minimal due to the severity of the adverse effects even if they only occur in a small number of cats.

In conclusion, at the plasma concentrations used, butorphanol resulted in a reduction in CI and oxygen delivery index and an increase in SVRI and oxygen consumption index in isoflurane-anesthetized cats, despite a reduction in isoflurane concentration. Due to the concurrent and opposite effects on CI and SVRI, MAP did not significantly change. Overall, these effects are considered to be detrimental.

## Data Availability

The raw data supporting the conclusions of this article will be made available by the authors, without undue reservation.

## Footnotes

^1^ http://random.org

## References

[1] Duke T. Partial intravenous anesthesia in cats and dogs. Can Vet J. (2013) 54:276–82. 23997266 PMC3573635

[2] Brosnan RJ Pypendop BH Siao KT Stanley SD. Effects of remifentanil on measures of anesthetic immobility and analgesia in cats. Am J Vet Res. (2009) 70:1065–71. doi: 10.2460/ajvr.70.9.1065, PMID: 19719420

[3] Brosnan RJ Pypendop BH Stanley SD. Phenylpiperidine opioid effects on isoflurane minimum alveolar concentration in cats. J Vet Pharmacol Ther. (2020) 43:533–7. doi: 10.1111/jvp.12886, PMID: 32557697

[4] Ilkiw JE Pascoe PJ Fisher LD. Effect of alfentanil on the minimum alveolar concentration of isoflurane in cats. Am J Vet Res. (1997) 58:1274–9. doi: 10.2460/ajvr.1997.58.11.1274, PMID: 9361892

[5] Ilkiw JE Pascoe PJ Tripp LD. Effects of morphine, butorphanol, buprenorphine, and U50488h on the minimum alveolar concentration of isoflurane in cats. Am J Vet Res. (2002) 63:1198–202. doi: 10.2460/ajvr.2002.63.1198, PMID: 12171176

[6] Ferreira TH Aguiar AJ Valverde A Neto FJ Steagall PV Soares JH. Effect of remifentanil hydrochloride administered via constant rate infusion on the minimum alveolar concentration of isoflurane in cats. Am J Vet Res. (2009) 70:581–8. doi: 10.2460/ajvr.70.5.581 19405896

[7] Hall RI Murphy MR Hug CC Jr. The enflurane sparing effect of sufentanil in dogs. Anesthesiology. (1987) 67:518–25. doi: 10.1097/00000542-198710000-00013 2959171

[8] Monteiro ER Teixeira-Neto FJ Campagnol D Alvaides RK Garofalo NA Matsubara LM. Effects of remifentanil on the minimum alveolar concentration of isoflurane in dogs. Am J Vet Res. (2010) 71:150–6. doi: 10.2460/ajvr.71.2.150, PMID: 20113221

[9] Pachter IJ Evens RP. Butorphanol. Drug Alcohol Depend. (1985) 14:325–38. doi: 10.1016/0376-8716(85)90065-1, PMID: 3888579

[10] Pypendop BH Goich M Shilo-Benjamini Y. Effect of intravenous butorphanol infusion on the minimum alveolar concentration of isoflurane in cats. Vet Anaesth Analg. (2022) 49:165–72. doi: 10.1016/j.vaa.2021.12.004, PMID: 35033447

[11] Copland VS Haskins SC Patz JD. Cardiovascular and pulmonary effects of atropine reversal of oxymorphone-induced bradycardia in dogs. Vet Surg. (1992) 21:414–7. doi: 10.1111/j.1532-950x.1992.tb01719.x, PMID: 1413473

[12] Pypendop BH Honkavaara J Ilkiw JE. Cardiovascular effects of dexmedetomidine, with or without Mk-467, following intravenous administration in cats. Vet Anaesth Analg. (2017) 44:52–62. doi: 10.1111/vaa.12397, PMID: 27377604

[13] Knych HK Casbeer HC McKemie DS Arthur RM. Pharmacokinetics and pharmacodynamics of butorphanol following intravenous administration to the horse. J Vet Pharmacol Ther. (2013) 36:21–30. doi: 10.1111/j.1365-2885.2012.01385.x 22339417

[14] Pypendop BH Shilo-Benjamini Y. Pharmacokinetics of butorphanol in male neutered cats anesthetized with isoflurane. J Vet Pharmacol Ther. (2021) 44:883–7. doi: 10.1111/jvp.13014, PMID: 34558086 PMC9293126

[15] Jaeger AT Pypendop BH Ahokoivu H Honkavaara J. Cardiopulmonary effects of dexmedetomidine, with and without vatinoxan, in isoflurane-anesthetized cats. Vet Anaesth Analg. (2019) 46:753–64. doi: 10.1016/j.vaa.2019.05.012 31416697

[16] de Jong RH Eger EI 2nd. Mac expanded: Ad50 and Ad95 values of common inhalation anesthetics in man. Anesthesiology. (1975) 42:384–9. doi: 10.1097/00000542-197504000-00003 235228

[17] Gross ME Smith JA Tranquilli WJ. Cardiorespiratory effects of combined midazolam and butorphanol in isoflurane-anesthetized cats. Vet Surg. (1993) 22:159–62. doi: 10.1111/j.1532-950x.1993.tb01692.x, PMID: 8511851

[18] Sederberg J Stanley TH Reddy P Liu WS Port D Gillmor S. Hemodynamic effects of butorphanol-oxygen anesthesia in dogs. Anesth Analg. (1981) 60:715–9. doi: 10.1213/00000539-198110000-00003, PMID: 7197474

[19] Trim CM. Cardiopulmonary effects of butorphanol tartrate in dogs. Am J Vet Res. (1983) 44:329–31. doi: 10.2460/ajvr.1983.44.02.329 6830022

[20] Simon BT Lizarraga I. Opioids In: Lamont LA Grimm KA Robertson S Love L Schroeder C, editors. Veterinary anesthesia and analgesia. Hoboken, NJ: Wiley Blackwell (2024). 355–97.

[21] Ilkiw JE Pascoe PJ Haskins SC Patz JD Jaffe R. The cardiovascular sparing effect of fentanyl and atropine, administered to enflurane anesthetized dogs. Can J Vet Res. (1994) 58:248–53. 7889455 PMC1263707

[22] Machado ML Soares JHN Pypendop BH Henao-Guerrero N Oliveira RLS. Effect of fentanyl, with or without treatment of bradycardia, on the minimum alveolar concentration of isoflurane and cardiovascular function in dogs. Vet Anaesth Analg. (2022) 49:26–35. doi: 10.1016/j.vaa.2021.09.001, PMID: 34654643

[23] Pascoe PJ Ilkiw JE Fisher LD. Cardiovascular effects of equipotent isoflurane and alfentanil/isoflurane minimum alveolar concentration multiple in cats. Am J Vet Res. (1997) 58:1267–73. doi: 10.2460/ajvr.1997.58.11.1267, PMID: 9361891

[24] Commiskey S Fan LW Ho IK Rockhold RW. Butorphanol: effects of a prototypical agonist-antagonist analgesic on kappa-opioid receptors. J Pharmacol Sci. (2005) 98:109–16. doi: 10.1254/jphs.crj05001x 15942128

[25] Volpe DA McMahon Tobin GA Mellon RD Katki AG Parker RJ Colatsky T. Uniform assessment and ranking of opioid μ receptor binding constants for selected opioid drugs. Regul Toxicol Pharmacol. (2011) 59:385–90. doi: 10.1016/j.yrtph.2010.12.007 21215785

[26] DeFrancesco TC. Management of cardiac emergencies in small animals. Vet Clin North Am Small Anim Pract. (2013) 43:817–42. doi: 10.1016/j.cvsm.2013.03.012 23747262

[27] Garofalo NA Teixeira Neto FJ Pereira CD Pignaton W Vicente F Alvaides RK. Cardiorespiratory and neuroendocrine changes induced by methadone in conscious and in isoflurane anaesthetised dogs. Vet J. (2012) 194:398–404. doi: 10.1016/j.tvjl.2012.03.019 22750283

[28] Maiante AA Teixeira Neto FJ Beier SL Corrente JE Pedroso CE. Comparison of the cardio-respiratory effects of methadone and morphine in conscious dogs. J Vet Pharmacol Ther. (2009) 32:317–28. doi: 10.1111/j.1365-2885.2008.01042.x 19614836

[29] Kristensen K Christensen CB Christrup LL. The mu_1_, mu_2_, delta, kappa opioid receptor binding profiles of methadone stereoisomers and morphine. Life Sci. (1995) 56:PL45–50. doi: 10.1016/0024-3205(94)00426-s, PMID: 7823756

[30] Greene SA Hartsfield SM Tyner CL. Cardiovascular effects of butorphanol in halothane-anesthetized dogs. Am J Vet Res. (1990) 51:1276–9. doi: 10.2460/ajvr.1990.51.08.1276, PMID: 2386328

[31] Sheriff DD Zhou XP Scher AM Rowell LB. Dependence of cardiac filling pressure on cardiac output during rest and dynamic exercise in dogs. Am J Physiol. (1993) 265:H316–22. doi: 10.1152/ajpheart.1993.265.1.H316 8342648

[32] Samodelov LF Samodelov G Arndt JO. Isoflurane and total oxygen consumption in spontaneously breathing dogs in basal metabolic conditions. Anaesthesist. (1985) 34:184–90. PMID: 4003747

[33] Lynch JP Mhyre JG Dantzker DR. Influence of cardiac output on intrapulmonary shunt. J Appl Physiol. (1979) 46:315–21. doi: 10.1152/jappl.1979.46.2.315, PMID: 422447

[34] Roebel LE Cavanagh RL Buyniski JP. Comparative gastrointestinal and biliary tract effects of morphine and butorphanol (Stadol). J Med. (1979) 10:225–38. 43349

[35] Roth SH Schofield B Yates JC. Effects of atropine on secretion and motility in isolated gastric mucosa and attached muscularis externa from ferret and cat. J Physiol. (1979) 292:351–61. doi: 10.1113/jphysiol.1979.sp012855, PMID: 490363 PMC1280862

