# Supplementary material for: Target-controlled infusions of butorphanol worsen hemodynamics in isoflurane-anesthetized cats
Source: Front Vet Sci. 2025 Jun 3;12:1600753. doi: 10.3389/fvets.2025.1600753 (PMC12170508; doi:10.3389/fvets.2025.1600753)
Supplement: Supplementary file 1 [file Table_1.DOCX]

Supplementary Material

# ANOVA *P* values

| Variable | TPC effect | Time effect | TPC × time effect |
| --- | --- | --- | --- |
| HR (beats minute^-1^) | <0.0001 | 0.393 | 0.536 |
| MAP (mm Hg) | 0.6573 | 0.1562 | 0.9613 |
| CVP (mm Hg) | 0.0001 | 1 | 1 |
| MPAP (mm Hg) | 0.0062 | 0.3845 | 0.7808 |
| PAOP (mm Hg) | 0.6676 | 0.7884 | 0.6491 |
| Body temperature (˚C) | 0.7001 | 0.1844 | 0.5989 |
| Arterial pH | 0.0113 | 0.0024 | 0.0425 |
| PaO_2_ (mm Hg) | 0.714 | 0.1614 | 0.167 |
| PaCO_2_ (mm Hg) | 0.2146 | 0.0362 | 0.0137 |
| Arterial HCO_3_^-^ (mEq L^-1^) | 0.008 | 0.9354 | 0.8558 |
| Arterial SBE (mEq L^-1^) | 0.0177 | 0.558 | 0.8932 |
| SaO_2_ (%) | 0.9175 | 0.3473 | 0.334 |
| MV pH | 0.0015 | 0.0361 | 0.1834 |
| PvO_2_ temp (mm Hg) | <0.0001 | 0.5465 | 0.7181 |
| PvCO_2_ (mm Hg) | 0.0661 | 0.3322 | 0.1581 |
| MV HCO_3_^-^ (mEq L^-1^) | 0.7469 | 0.2743 | 0.2679 |
| MV SBE (mEq L^-1^) | 0.2961 | 1 | 0.8882 |
| SvO_2_ (%) | <0.0001 | 0.8326 | 0.4722 |
| CI (L minute^-1^ BW^-0.67^) | <0.0001 | 0.9361 | 0.052 |
| SI (mL beat^-1^ kg^-1^) | 0.0004 | 0.6438 | 0.0327 |
| SVRI (dynes seconds cm^-5^ BW^-0.67^) | 0.0087 | 0.2435 | 0.3658 |
| PVRI (dynes seconds cm^-5^ BW^-0.67^) | 0.0645 | 0.9717 | 0.6851 |
| LVSWI (cJ kg^-1^) | 0.0327 |  | 0.6345 |
| RVSWI (cJ kg^-1^) | <0.0001 | 0.2153 | 0.777 |
| RPP (beats minute^-1^ mm Hg) | 0.0262 | <0.0001 | 0.9907 |
| CaO_2_ (mL dL^-1^) | 0.0072 | 0.322 | 0.9616 |
| CvO_2_ (mL dL^-1^) | <0.0001 | 0.071 | 0.3639 |
| DO_2_I (mL minute^-1^.BW^-0.67^) | <0.0001 | 0.3955 | 0.2522 |
| VO_2_I (mL minute^-1^.BW^-0.67^) | 0.0022 | 0.5628 | 0.0952 |
| Oxygen utilization ratio^a^ | <0.0001 | 0.391 | 0.1461 |
| Qs/Qt | <0.0001 | 0.1991 | 0.1225 |
| P(A-a)O_2_ (mm Hg) | 0.6948 | 0.1458 | 0.1476 |

TPC: target plasma concentration; HR: heart rate; MAP: mean arterial pressure; CVP: central venous pressure; MPAP: mean pulmonary artery pressure; PAOP: pulmonary artery occlusion pressure; PaO_2_: arterial partial pressure of oxygen; PaCO_2_: arterial partial pressure of carbon dioxide; HCO_3_^-^: plasma bicarbonate concentration; SBE: standard base excess; SaO_2_: arterial hemoglobin oxygen saturation; MV: mixed venous; PvO_2_: mixed venous partial pressure of oxygen; PvCO_2_: mixed venous partial pressure of carbon dioxide; SvO_2_: mixed venous hemoglobin oxygen saturation; CI: cardiac index; BW: body weight; SI: stroke index; SVRI: systemic vascular resistance index; PVRI: pulmonary vascular resistance index; LVSWI: left ventricular stroke work index; RVSWI: right ventricular stroke work index; RPP: rate-pressure product; CaO_2_: arterial oxygen concentration; CvO_2_: mixed venous oxygen concentration; DO_2_I: oxygen delivery index; VO_2_I: oxygen consumption index; Qs/Qt: shunt fraction; P(A-a)O_2_: alveolar-to-arterial difference in partial pressure of oxygen.

# Pairwise target plasma butorphanol comparisons

| Variable | 0 *vs* 1.1 µg mL^-1^ | 0 *vs* 2.2 µg mL^-1^ | 1.1 *vs* 2.2 µg mL^-1^ |
| --- | --- | --- | --- |
| HR (beats minute^-1^) | 0.0023 | <0.0001 | 0.003 |
| CVP (mm Hg) | 0.1605 | 0.0001 | 0.0024 |
| MPAP (mm Hg) | 0.7116 | 0.0049 | 0.202 |
| Arterial pH | 0.0643 | 0.0101 | 0.4835 |
| Arterial HCO_3_^-^ (mEq L^-1^) | 0.7538 | 0.0085 | 0.0272 |
| Arterial SBE (mEq L^-1^) | 0.3317 | 0.016 | 0.0702 |
| MV pH | 0.0364 | 0.0012 | 0.0749 |
| PvO_2_ temp (mm Hg) | 0.0045 | <0.0001 | 0.0004 |
| SvO_2_ (%) | 0.1018 | <0.0001 | 0.0004 |
| CI (L minute^-1^ BW^-0.67^) | 0.0009 | <0.0001 | 0.0004 |
| SI (mL beat^-1^ kg^-1^) | 0.1583 | 0.0004 | 0.0051 |
| SVRI (dynes seconds cm^-5^ BW^-0.67^) | 0.1595 | 0.0072 | 0.0767 |
| LVSWI (cJ kg^-1^) | 0.8619 | 0.0367 | 0.0678 |
| RVSWI (cJ kg^-1^) | 0.2748 | <0.0001 | 0.0004 |
| RPP (beats minute^-1^ mm Hg) | 0.1824 | 0.0056 | 0.1558 |
| CaO_2_ (mL dL^-1^) | 0.6933 | 0.0237 | 0.0081 |
| CvO_2_ (mL dL^-1^) | 0.0003 | <0.0001 | 0.0008 |
| DO_2_I (mL minute^-1^.BW^-0.67^) | 0.0006 | <0.0001 | 0.0016 |
| VO_2_I (mL minute^-1^.BW^-0.67^) | 0.061 | 0.0017 | 0.0767 |
| Oxygen utilization ratio^a^ | 0.0038 | <0.0001 | 0.0005 |
| Qs/Qt | 0.0084 | <0.0001 | 0.0061 |

Pairwise TPC comparisons were only conducted if the target plasma concentration effect in the ANOVA was significant. See ANOVA table for key.

# Time comparisons (20 *vs* 45) within target plasma butorphanol concentration

| Variable | 0 µg mL^-1^ | 1.1 µg mL^-1^ | 2.2 µg mL^-1^ |
| --- | --- | --- | --- |
| Arterial pH | 0.0449 | 0.3972 | 0.207 |
| PaCO_2_ (mm Hg) | 0.0745 | 0.1061 | 0.9419 |
| MV pH | 0.1835 | 0.5624 | 0.0776 |
| RPP (beats minute^-1^ mm Hg) | 0.0377 | 0.0743 | 0.1806 |

Time comparisons within target plasma concentration were only conducted if the time effect in the ANOVA was significant. See ANOVA table for key.
